# Integration and Analysis of Neighbor Discovery and Link Quality Estimation in Wireless Sensor Networks

**DOI:** 10.1155/2014/789642

**Published:** 2014-02-11

**Authors:** Marjan Radi, Behnam Dezfouli, Kamalrulnizam Abu Bakar, Shukor Abd Razak

**Affiliations:** Faculty of Computing, Universiti Teknologi Malaysia, Johor 81310, Malaysia

## Abstract

Network connectivity and link quality information are the fundamental requirements of wireless sensor network protocols to perform their desired functionality. Most of the existing discovery protocols have only focused on the neighbor discovery problem, while a few number of them provide an integrated neighbor search and link estimation. As these protocols require a careful parameter adjustment before network deployment, they cannot provide scalable and accurate network initialization in large-scale dense wireless sensor networks with random topology. Furthermore, performance of these protocols has not entirely been evaluated yet. In this paper, we perform a comprehensive simulation study on the efficiency of employing adaptive protocols compared to the existing nonadaptive protocols for initializing sensor networks with random topology. In this regard, we propose adaptive network initialization protocols which integrate the initial neighbor discovery with link quality estimation process to initialize large-scale dense wireless sensor networks without requiring any parameter adjustment before network deployment. To the best of our knowledge, this work is the first attempt to provide a detailed simulation study on the performance of integrated neighbor discovery and link quality estimation protocols for initializing sensor networks. This study can help system designers to determine the most appropriate approach for different applications.

## 1. Introduction

In large-scale wireless sensor networks, hundred or thousand nodes are usually deployed in an ad hoc fashion without any predefined infrastructure [1]. For instance, configuration of a wireless sensor network for a disaster management application in an inaccessible area requires ad hoc deployment of a large number of sensor nodes by dropping from a helicopter. Therefore, neighbor discovery is a fundamental step to initialize wireless sensor networks and provide individual nodes with initial information regarding their immediate neighboring nodes [2–4]. This information is essential for various higher layer protocols such as Medium Access Control (MAC) [5], scheduling algorithms [6], collection tree [7], routing [8, 9], and localization protocols [10] to execute correctly and efficiently. For instance, Carrier Sense Multiple Access- (CSMA-) based MAC protocols can use neighborhood information of network nodes to select a set of nodes that can be activated at the same time while their concurrent activation results in a minimum number of collisions [11, 12]. In addition, the employed scheduling algorithms in TDMA-based MAC protocols utilize two-hop neighborhood information of individual nodes as a basis for scheduling the available time slots so that no two interfering nodes access the channel at a same time [13]. Furthermore, due to the short communication range of low-power radio transceivers, network nodes always perform multihop communications for data dissemination. In view of that, routing and collection tree protocols require the preserved neighborhood information at individual nodes to construct the network routing zone and provide efficient data dissemination [14].

Since high dynamics and time-varying properties of wireless communications cause unpredictable communication links between different nodes, performing neighbor discovery alone is not enough to support robust network connectivity for a long time [15, 16]. High variations of data transmission quality over wireless links have been empirically shown through various real-world implementations with different platforms and experimental conditions [17, 18]. Based on these studies, network nodes should be aware about the condition of available links towards their neighboring nodes in order to construct energy-efficient and reliable network topology [19]. Energy-efficient and reliable data transmission can be achieved through data transmission over reliable links, which require a few number of retransmissions for successful packet delivery [20–22]. In this context, several link quality measurement techniques have been proposed over the past decade. These mechanisms utilize different link quality indicators such as Packet Reception Ratio (PRR), Received Signal Strength Indicator (RSSI), and Link Quality Indicator (LQI) to provide link quality estimations during network operation [23–25]. Through performing extensive studies on the impacts of link quality assessment on the performance of network protocols, link quality estimation has been considered as a fundamental building block of communication protocols [15]. For example, routing protocols can exploit the link layer information to overcome the unreliability of wireless links and provide stable network performance [26, 27]. In other words, data transmission over high-quality paths improves network throughput and network lifetime by reducing packet loss ratio and frequency of route reconstruction process. Moreover, to maintain a stable network performance, topology control protocols can use link quality information to construct initial network topology through long-lived links. Therefore, it is valuable to collect initial information regarding availability and data transmission quality of wireless links through an integrated neighbor discovery and link estimation technique prior to the network operation phase.

To the best of our knowledge, only a limited number of integrated neighbor discovery and link estimation protocols have been proposed to enable network nodes to collect initial information regarding their neighboring nodes along with their respective data transmission quality upon their deployment [28, 29]. These protocols perform link assessment process while the network topology is discovered through assessing a predefined number of beacon messages exchanged between each neighbor pair. In this regard, during network initialization all the nodes broadcast a specific number of beacon messages (e.g., 10 beacon messages) with a predefined beaconing interval (e.g., every 1 second) to measure data transmission quality of their incoming and outgoing links. Since network initialization phase uses CSMA for channel arbitration, and this phase includes a lot of broadcast transmissions to perform neighbor discovery and link quality measurement process [27, 29], these protocols cannot adaptively provide accurate neighbor discovery and link estimation in large-scale wireless sensor networks with random deployments. The reason can be explained as follows. A perfect network initialization protocol which integrates the neighbor discovery process with link estimation should be able to distinguish between packet losses caused by collision from those caused by poor link connectivity. Accordingly, network initialization protocols should accurately detect the neighboring nodes of every node and estimate data transmission quality of available links based on the path loss and system variations. However, due to the broadcast nature of wireless communications, the accuracy of these protocols which utilize constant beaconing rate at all the nodes highly depends on the precision of beaconing rate adjustment at individual nodes before network deployment. Since these protocols require a careful parameter adjustment before network deployment, they cannot provide scalable and accurate neighbor discovery and link estimation in large-scale dense wireless sensor networks with unknown topology or uneven node density. In [28], collision-free beacon exchange at the network initialization phase is provided through several assumptions such that nodes are synchronized to each other, and every node knows the maximum number of its neighbors before network deployment. In addition to the negative effects of packet collisions, the beacon transmission sequence of neighboring nodes of individual nodes has a high impact on the accuracy of network initialization protocols. In fact without supporting an interleave beacon exchange pattern between nodes, it is highly possible that some nodes finalize their broadcasts before receiving the entire transmitted beacon messages by their neighbors. As a consequence, the nodes which have completed the beacon transmission process before their neighboring nodes cannot provide their neighbors with accurate link quality information.

Although several neighbor discovery protocols and link quality estimation approaches have been developed over the past decade, integration of initial neighbor discovery and link quality estimation as a separated phase has not been subject of a comprehensive evaluation yet. Therefore, the major contributions of this paper are as follows.

- In order to highlight the drawbacks of employing existing nonadaptive network initialization protocols in dense wireless sensor networks with unknown topology through comprehensive performance evaluations, we propose an Adaptive Network Initialization protocol with Single Beaconing approach (ANI-SB) which integrates the initial neighbor search with a link quality estimation technique. The aim of this protocol is to allow nodes to adjust the time interval between their beacon transmissions according to their neighborhood size. The first goal of this beaconing rate adjustment is to reduce the effects of packet collisions on the accuracy of achieved neighborhood information by the network nodes. The second goal is to provide an interleave beacon exchange pattern between neighboring nodes to improve the accuracy of link estimations. In fact, as this method enables nodes to update the experienced PRR from their identified neighboring nodes before broadcasting the next beacon message, all the nodes can be informed about the updates on the PRR of their outgoing and incoming links. Moreover, same as the existing network initialization protocols [7, 29, 30], through this protocol every node only broadcasts a single beacon message in each beaconing round. Therefore, like other protocols, whenever a node could not include its whole neighborhood information in a single beacon message, it selects a subset of its identified nodes to broadcast their related information.
- In order to study the effects of sharing the entire neighborhood information of every node at each beacon transmission round instead of selecting the related information of only a subset of neighbors in dense wireless sensor networks, we improve the ANI-SB protocol through proposing an Adaptive Network Initialization protocol with Multiple Beaconing approach (ANI-MB). This protocol aims to enable nodes in high-density networks to announce their whole neighborhood information at every beacon transmission round through multiple consecutive beacons, whenever the available space in a single packet is not enough to share their entire neighborhood information. The main goal of this protocol is to improve the accuracy of initial neighbor discovery and link estimation process in large-scale dense wireless sensor networks.
- We perform a comprehensive simulation study on the capability of the existing nonadaptive and proposed adaptive network initialization protocols in providing efficient network initialization in large-scale dense wireless sensor networks. In the first part of simulation studies, we analyze the performance of different network initialization protocols in one-hop topology networks, and then in the second part we study the effects of multihop topology on the performance network initialization protocols.

The rest of the paper is organized as follows.  Section 2 presents the background of this research. The main challenges in developing integrated neighbor discovery and link quality estimation protocols are highlighted in Section 3.  Section 4 describes the main operation of the considered nonadaptive and proposed adaptive network initialization protocols for performance evaluation studies. The simulation model is introduced in Section 5. The considered network initialization protocols are analyzed and compared in Section 6. Finally, some conclusion remarks are given in Section 7.

## 2. Background

This section gives an overview on the previous works, which have been done on neighbor discovery, combination of link quality estimation with initial neighbor discovery and performance evaluation studies on the existing neighbor discovery and link quality estimation protocols.

### 2.1. Neighbor Discovery

Over the past decade, several neighbor discovery protocols have been developed to discover initial network connectivity and identify topological changes during network operation [2, 3]. Birthday Protocol is the first asynchronous energy-efficient discovery protocol which uses a randomized mechanism for enabling nodes to transit between sleep, listen, and transmission states during the neighbor discovery process [31]. Although using probabilistic approaches for changing nodes' state from transmit to listen or sleep mode can reduce the network energy consumption, it will yield unpredictable neighbor discovery latency. Another asynchronous probabilistic neighbor discovery algorithm is presented in [32]. In this protocol, nodes should have an accurate estimation on the number of their neighboring nodes before initializing neighbor discovery to calculate the probability of beacon transmission at each slot. Since all the parameters are determined based on a fixed node density, this algorithm cannot adaptively work in irregular densities. To support power saving modes for IEEE 802.11-based Mobile Ad hoc Networks (MANETs) through scheduling the wake-up time of each pair of nodes, Quorum protocol is proposed in [33]. This algorithm adapts the quorum concept to develop wake-up schedule of the mobile hosts so that the transmitted packets by a mobile host can always be received by the other hosts. The main application of this protocol is to maintain network connectivity during network operation. Disco is also designed based on the Chinese Reminder Theorem to schedule wake-up times of node pairs and provide continuous neighbor discovery [34]. In fact, the focus of this protocol is on preventing long period of disconnections even in the mobile networks while it supports low-duty cycle network operation. An Energy-efficient Neighbor Discovery Protocol (ENDP) for synchronized low-duty cycle MAC protocols (e.g., S-MAC [35] and IEEE 802.15.4 [36]) in low-power mobile networks is designed in [37]. In order to avoid packet collisions, ENDP assumes that the transmission interval of beacon messages is longer than several orders of a single network beacon transmission and it also exploits a multichannel approach for broadcasting beacon messages. In addition, as ENDP works with synchronized MAC protocols and nodes utilize the payload of the existing MAC beacons for distributing neighborhood information, the beacon transmissions are synchronized by the MAC protocol. Accordingly, ENDP provides energy-efficient and accurate neighbor discovery through employing a synchronized MAC protocol and a multichannel transmission technique.

### 2.2. Integration of Neighbor Discovery with Link Quality Estimation

Several research works have studied the empirical characteristics of radio communications [16]. All of these studies have confirmed the unpredictable behavior of radio communications and high fluctuations of data transmission quality over wireless links. According to these observations, link quality estimation is known as a fundamental building block of different network protocols in wireless sensor networks, and various link assessment techniques are designed to improve network performance [15, 38]. Although there exist several research works which have introduced various neighbor discovery mechanisms and rich literature on the link quality estimation in wireless networks, still integration of initial neighbor discovery and link quality estimation has a lot of open research challenges [2]. In fact, the combination of neighbor discovery and link quality estimation as a separate phase to provide preliminary information regarding the available network links and their respective data transmission performance has not attracted enough attention [2, 28, 29].

A link assessment process for a combination of neighbor discovery and link grading is proposed in [28]. Authors have suggested constant-weight codes can provide energy-efficient neighbor discovery and link quality measurement in a slotted structure. This protocol provides energy-efficient communications through assuming nodes are synchronized to each other, and each node can be in one of the sleep, transmit, and receive modes at each time slot. In this approach, every node calculates the required number of collision-free slots and probability of transiting to different states (i.e., sleep, transmit, and receive) in each slot, based on the maximum neighborhood size. Accordingly, accurate estimation of the maximum neighborhood size and node synchronization prior to network topology formation are essential for providing a good network performance. Furthermore, it is assumed that the number of neighboring nodes of each node and their respective link quality do not change during the estimation process. Although this approach can provide accurate neighbor discovery and link quality estimations during network initialization phase, it is impossible to achieve all of the considered assumptions in the real-world implementations. NoSE is another network initialization protocol which aims to conduct an integrated neighbor discovery and link quality assessment [29]. In this protocol sink node triggers the start of network initialization process through flooding a wake-up message into the network upon deployment of all the network nodes. Since the wake-up message includes essential information to perform neighbor discovery and link quality measurement (e.g., starting time of neighbor discovery, discovery duration, number of beacon messages, and beacon transmission rate), all the nodes should receive this message, before beginning the discovery phase. NoSE tries to yield a time-bounded network initialization by forcing nodes to divide the user-defined discovery duration into the subslots equal to the number of predefined beacon messages and then select a random transmission time for each of them. Moreover, all the nodes should maintain the number of received packets from their individual neighboring nodes and maximum RSSI value of the transmitted packets to estimate data transmission quality of available links. In this method, the asymmetric property of low-power links has not been considered well, because individual nodes only calculate the PRR of their incoming links. Furthermore, NoSE uses the available information regarding the RSSI value of the received packets to preserve neighborhood information of the best neighboring nodes for each node. By this technique, each node only includes the neighboring nodes with PRR more than 90% in its neighborhood table. However, PRR of a large number of links in large-scale wireless sensor networks varies between 10% and 90% [39, 40]. As most of the network protocols, such as MAC, scheduling, and routing protocols require a complete list of neighboring nodes at individual nodes to perform their functionality [5, 9], this approach cannot be used to provide the required basic information for a wide range of protocols. Furthermore, this protocol uses a carrier sensing approach to reduce the probability of packet collisions. Even by using the carrier sensing mechanism, still there is a possibility for packet collisions due to the hidden node terminal problem [11, 12]. Accordingly, to avoid these collisions the discovery period should be determined based on the maximum neighborhood size before network deployment so that the channel provides a large bandwidth. However, in some applications nodes are distributed in the area of interest without any predetermined network infrastructure and neighborhood density of individual nodes cannot be easily estimated before network initialization phase [41, 42].

### 2.3. Performance Evaluation of Neighbor Discovery and Link Quality Estimation Protocols

In the context of performance evaluation and comparison studies, the performance of integrated neighbor discovery and link estimation protocols is not thoroughly evaluated yet. Galluzzi and Herman give an overview on the neighbor discovery in wireless sensor networks without considering link quality measurement issues at the initialization phase [3]. The main purpose of their work was to present some background concepts on the wake-up problem in neighbor discovery and a brief comparison study on the discovery length of a number of existing neighbor discovery protocols (i.e., Birthday protocol [31], Quorum [33], Disco [34], and U-connect protocol [43]). The presented comparison study is restricted to comparing the discovery latency of the existing protocols, which have been developed to handle the neighbor discovery problem without paying attention to the link layer issues. The impacts of collisions and wireless interference on the performance of neighbor discovery process are investigated in [44]. The authors analytically modeled the link success probability and average number of nodes that can correctly receive a beacon in three radio channel models. However, it is restricted to modeling the packet transmission over radio links according to the utilized method to handle collisions and interference, while the authors have not analyzed the efficiency of integrating initial neighbor discovery with link estimation process during initialization of large-scale wireless sensor networks.

There also exist a number of works on the performance evaluation and comparison of the existing link quality measurement metrics [19, 23, 45]. In all of these works, two evaluation methodologies are considered to analyze the performance of various link quality estimation approaches. The key objective of using the first methodology was to analyze the statistical properties of link quality estimators independent of higher layer protocols while the second methodology aims to focus on assessing the performance of existing link quality measurement metrics in combination with routing protocols. Through these methodologies, the existing comparative studies investigate about the performance of various link quality metrics and their impacts on the efficiency of the routing protocols. Nevertheless, none of these studies have evaluated the efficiency of integrating the initial neighbor discovery with the link quality assessment process to provide all the nodes with a comprehensive list of their neighboring nodes along with their respective data transmission quality during the network initialization phase.

## 3. Challenges in Integration of Initial Neighbor Discovery and Link Quality Estimation

Since, upon deployment of a network, installed nodes do not have any information about the other existing nodes in the network, accurate initial neighbor discovery and link quality assessment are a challenging task. Moreover, complex and dynamic behavior of low-power wireless links makes neighbor discovery and link quality estimation process even harder [15, 46].

**Figure 1:**
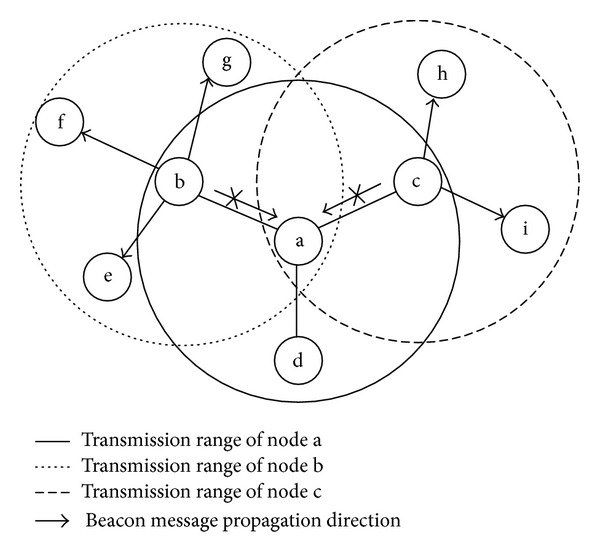
Impact of packet collisions on the accuracy of neighbor discovery and link estimation process.

During the network initialization phase, a predefined number of collision-free beacon messages should be exchanged between each pair of neighbors, to conduct a successful link quality assessment while the nodes are being discovered. A collision can take place if at least two nodes transmit their packets concurrently to a same node which is a common neighbor for all of these transmitters. This situation occurs if two nodes select a same time slot in a contention window or due to the hidden terminal problem [47]. As, during collision, the receiver node cannot receive any of the transmitted packets correctly, initialization protocols should minimize the probability of collisions to provide accurate neighborhood information at individual nodes.  Figure 1 shows this issue with a simple example. According to this figure, node b and node c are invisible to each other, but both of them are in the transmission range of node a. Since node a is a common neighbor for node b and node c, concurrent beacon broadcasts by these two nodes cause collision at node a and it cannot receive the transmitted beacon messages by these nodes. As, in the real-world deployments, nodes do not have prior knowledge about their vicinity and they are not time synchronized upon their installation, it is extremely hard to provide collision-free beacon transmissions at the network initialization. Moreover, as at the network initialization phase there is no active traffic to provide link quality information at different nodes, active link probing is the only way for measuring data transmission quality of network links. Therefore, all the nodes should broadcast a predefined number of beacon messages to measure the achievable PRR over different links. In addition whenever a node identifies new neighboring nodes, it should include the number of successfully received packets from these nodes into the next beacon messages to inform them about data transmission quality of their outgoing links. For instance, in Figure 1 node a should broadcast a fixed number of beacon messages to its neighboring nodes (i.e., node b, and c) in order to inform them about the PRR of their incoming links. All the neighboring nodes can estimate the feasible PRR over their incoming links from node a, by dividing the number of received packets from this node during the last time window by the total number of transmitted packets. In addition, nodes b and c should inform node a about the PRR over its outgoing links towards themselves. In view of that, node b and node c should include the number of successfully received packets from node a into their outgoing beacon messages in order to enable node a to estimate data transmission quality of its outgoing links (e.g., a-b and a-c). Therefore, missing beacon messages during network initialization process which is caused by collision will significantly affect the accuracy of link estimations and number of discovered nodes at individual nodes. Furthermore, without controlling the beaconing rate of network nodes, it is possible that a given node broadcasts all of its beacon messages before its neighbors finalize their beacon transmissions. Therefore, as this node would not transmission further beacon messages, its neighboring nodes cannot estimate the data transmission quality of their outgoing links correctly. For example if node b and node c in Figure 1 finalize their beacon transmissions before node a, they cannot inform node a about the exact number of its transmitted packets which have been successfully received by themselves. Consequently, node a could not update data transmission quality of its outgoing links (e.g., a-b, and a-c) correctly. According to this example, providing an interleave beacon transmission pattern between nodes plays an important role in providing accurate link estimations. In the existing protocols, all the nodes should send beacon messages with a fixed interval (e.g., every one second) within a predefined time window (e.g., 10 seconds) to reduce collisions and provide an interleave beacon exchange pattern, while it also yields a deterministic neighbor discovery duration [28–30, 48, 49]. However, since random deployment of network nodes may result in nonuniform network density, determining a constant beaconing rate for all the nodes before network deployment cannot provide adaptive solutions in the cases where no exact network density can be found for large-scale wireless sensor networks with random deployment (e.g., when sensor nodes are dropped from a helicopter). This is due to the fact that the number of packet collisions during this phase is directly related to the beaconing rate of the nodes and network density. In order to exclude the effects of packet collisions on the accuracy of neighbor discovery and link measurement process through forcing the entire nodes to broadcast beacon messages at a fixed rate, beaconing interval of the nodes should be determined based on the their neighborhood size before network deployment so that the channel provides a large bandwidth. Since the existing protocols require exact parameter adjustment by the system engineer before network deployment, they cannot be used in the cases where the network topology is unknown.

Furthermore, PRR over wireless links highly depends on the size of transmitted packets [50]. In fact, probability of receiving error-free packets is related to the packet size. With a given bit error rate, there is a less probability that small size packets are more affected by wireless interference than the large packets [51]. During the initialization phase, whenever a node identifies new neighboring nodes, it should include the number of received packets from these nodes into the next beacon messages to inform them about the PRR over their outgoing links. Therefore, if network nodes of dense wireless networks with nonuniform node density want to share their whole neighborhood information, they may broadcast beacon messages with different sizes. However, broadcasting beacon messages with various sizes cannot provide accurate estimations on the PRR of different links.

## 4. Network Initialization Protocols under Evaluation

To study the performance of integrated neighbor discovery and link quality estimation protocols, we consider three network initialization variants. In the first variation, the same as the existing protocols [23, 29, 30, 48] each node broadcasts a predefined number of beacon messages at a certain rate (i.e., one beacon every one second) to perform initial link estimations. However, due to the infrastructureless nature of wireless sensor networks, it is almost impossible to determine the optimal beaconing rate of individual nodes before network deployment. Accordingly, as the second and third variations we introduce two adaptive protocols which aim to provide accurate and scalable network initialization through adjusting the beaconing rate of individual nodes based on their neighborhood density. The rest of this section describes the detailed operation of these protocols which are considered to evaluate and compare performance of the adaptive and nonadaptive integrated neighbor discovery and link estimation protocols.

### 4.1. An Integrated Neighbor Discovery and Link Estimation Protocol with Constant Beaconing Rate Adjustment

In the first variant which is called Constant Neighbor discovery and link Estimation (CNE) in this paper, all the nodes intensively participate in the neighbor discovery and link quality measurement process upon network deployment through broadcasting a predefined number of beacon messages at a fixed rate (e.g., one beacon/second) and listen to the channel to explore their neighborhood [23, 29, 30, 48]. At each time frame, a node selects a random start time to broadcast a single beacon message. Furthermore, all the nodes perform carrier sensing before initiating a new broadcast to reduce the probability of packet collisions due to the concurrent transmissions. Upon reception of a beacon message from a given node, the receiver node fetches the identity of the transmitter from the received message and searches its neighborhood table to see if the sender was identified so far. If the receiver node could not find the identity of the sender node in its neighboring table, it adds an entry in its neighboring table to keep the related information about this newly identified neighboring node. Each node also preserves the number of received beacon messages from all of its identified neighboring nodes. At the end of network initialization phase, this information enables the nodes to measure the PRR of their incoming links from the identified neighbors through counting the number of successfully received packets and remembering the number of beacon messages that should have been received. Furthermore, whenever a node wants to broadcast a new beacon message, it includes the number of received packets from its neighboring nodes during the last seconds. Distribution of this information allows all the nodes to inform their neighbors about data transmission quality of their outgoing links. In this approach, the network initialization phase ends when the entire nodes broadcast all of the predefined number of beacon messages.

### 4.2. Integrated Neighbor Discovery and Link Estimation Protocols with Adaptive Beaconing Rate Adjustment

Since during network initialization phase a lot of beacon messages are being broadcasted, the probability of packet collision is extremely high. Moreover, as the channel load depends on the minimum length of neighbor discovery and link quality assessment process, choosing inappropriate time duration for network initialization can lead to further increases in the probability of message collisions. To overcome these problems, we proposed the ANI-SB which aims to provide an adaptive beacon rate adjustment during the network initialization phase based on the neighborhood density of the nodes. This beaconing rate adjustment provides two goals. Hidden terminal avoidance and collision reduction during the broadcasts are the first goal, and affording enough time for one-hop neighboring nodes of each node to broadcast their beacons before it broadcasts a new beacon message is another goal. In fact, by this mechanism there is less probability that a node finishes its beacon transmission before it has received all the predefined beacons from its neighboring nodes. Accordingly, as the network initialization phase proceeds, every node can adjust its beaconing rate based on the beaconing rate of its neighbors.

**Figure 2:**
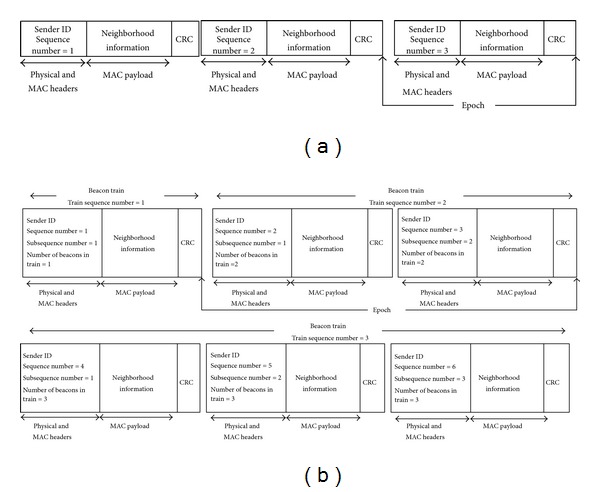
(a) Single-beaconing approach. (b) Multiple-beaconing approach.

Same as the CNE protocol, at the start of network initialization phase all the nodes perform neighbor discovery and link quality measurement process through broadcasting beacon messages periodically. Since, at the start of network initialization, nodes do not have any information regarding the structure of their vicinity, they should broadcast their first beacon message as soon as possible to announce their presence. In order to adjust the beaconing interval of the individual nodes, in ANI-SB every node initializes its beaconing rate upon reception of the first beacon message from one of its neighboring nodes according to time difference between the time it starts the network initialization process and the reception time of the first received beacon message from that particular neighboring node. Therefore, each node waits before transmitting the second and subsequent beacon messages based on the estimated beaconing intervals for its identified neighboring nodes, and then it broadcasts a new beacon message after performing a carrier sensing. By this mechanism, each node waits for receiving beacon messages from its neighboring nodes based on the maximum estimated beaconing interval for the neighboring nodes it has identified so far. The time interval between beacon transmissions of a node is called an epoch in the proposed protocol. During the network initialization process, whenever a node receives a new beacon message from one of its identified neighbors, it should update its waiting time for this neighbor according to reception time of the last and just received beacon messages from that particular neighbor. In the case of successive beacon losses during broadcasts of a given node, its neighbors are still able to update their waiting time upon complete reception of the next successfully transmitted beacon. For example, assume one of the neighboring nodes of the considered node in the Figure 2(a) did not receive the second beacon message. Upon reception of the third beacon message, this neighbor realizes that the second beacon is lost through checking the beacon sequence number of the received packet. Therefore, this node can estimate the beacon interval of its neighboring node by identifying the number of lost beacons and the time interval between last received beacon massage and complete reception of the current beacon message.

In order to perform link quality estimation, all the nodes preserve the number of received packets from every single node which is added to their neighborhood table. Same as the CNE protocol, individual nodes calculate the PRR over their incoming links through dividing the number of received packets from each neighboring node by the total number of broadcasted beacon messages by that node. In view of that, upon reception of a broadcast message, the receiver node updates the number of received packets from the sender of this message according to the sequence number of the received beacon message. In order to allow the nodes to calculate the PRR of their outgoing links, each node includes the number of received beacon messages from its neighboring nodes in its beacon message when it wants to broadcast a new beacon message. Therefore, whenever a given node wants to broadcast a new beacon message, it should prepare this message based on the last updated values for the number of received packets from individual neighboring nodes. Since a node may receive new beacon messages from its neighboring nodes during its bakeoff time, in the proposed protocol all the nodes prepare their beacon messages immediately before packet transmission. Thus, they can update the number of received packets from different neighboring nodes based on the last received packets and distribute the last updates regarding the PRR over outgoing links of their neighboring nodes.

Although broadcasting beacon messages with a same size to the data packets can ensure that all the links have been estimated under the same condition as the network operation phase, in the random topology individual nodes may a have different number of neighbors. Consequently, the nodes with high neighborhood density could not be able to fit their neighborhood information in a single beacon message. In fact, as in large-scale wireless sensor networks, every 3 bytes of each single beacon packet (i.e., 2 bytes for identity of a given node and 1 byte for the number of received beacons from the corresponding node) should be allocated to share the related information of a single node and the payload size of TinyOS packets is 29 bytes, each beacon message can include the identity and data transmission quality of up to 9 nodes. Therefore, even by utilizing beacon packets with equal size to the actual data packets, individual nodes of a large-scale wireless sensor network with high node density are still unable to share their entire neighborhood information in every beacon transmission round. Accordingly, by broadcasting a single fixed size beacon message some of the nodes would not be able to update the PRR of their outgoing links through all of the broadcasted beacons while broadcasting beacon messages with various sizes cannot provide valid and fair link quality estimations. In the existing protocols whenever the available space in a single beacon message is not sufficient to include the related information of the entire identified neighbors, the sender node selects a subset of its neighboring nodes to include their relative information in the beacon message through performing a round robin procedure [7, 29, 30]. Since by employing these protocols every transmitted beacon message during network initialization process would not enable all of the nodes to update the achievable PRR over their outgoing links, missing a single beacon packet caused by collision highly influences the accuracy of link estimations and number of discovered nodes by every node. In order to investigate about this issue, we propose ANI-MB protocol which enables the nodes to broadcast multiple beacons in a beacon transmission round when the neighborhood density of a node is more than a threshold. Under this mechanism, when the neighboring information of a given node cannot be fitted in a single beacon message, it is allowed to include the whole neighborhood information in multiple beacons and broadcast them consecutively. As the transmission of a single beacon is replaced with multiple beacons, which included the entire neighborhood information of a single node, the proposed protocol calls these multiple beacons as a beacon train. Since individual nodes may have different neighborhood densities, each node may broadcast various number of beacons in a beacon train. Therefore, every node should be aware about the number of beacons that each of its neighboring nodes wants to broadcast in order to calculate the beaconing interval of that particular neighboring node. In view of that, each transmitted beacon message in a beacon train includes the number of transmitted beacons in that particular beacon train as well as an identical subsequence number which identifies the order of the beacon in that specific beacon train. According to Figure 2(b), every beacon message which is transmitted as a single beacon or multiple beacons in beacon trains has a unique subsequence number in individual beacon trains, while each one has a global sequence number to identify the sequence of all the transmitted beacon messages by every node. Upon reception of a new beacon train, the receiver node can calculate the reception time of the whole current beacon train based on the number of included beacons in that particular beacon train and transmission duration of every beacon (i.e., the ratio of the beacon size to the radio bit rate). Therefore, the receiver node can update the beacon train transmission interval of the sender node according to the reception time of the whole current beacon train and the reception time of the last received train from that specific node. Furthermore, every node that receives a new beacon train should also update the number of received beacon trains from the transmitter node based on the beacon train sequence number of the received train to calculate its corresponding PRR. Although every node may transmit different number of beacons in a beacon train, still nodes can estimate beaconing interval of their neighbors even in the case of packet losses through included information in every transmitted beacon message. Assumes one of the neighboring nodes of the considered node in Figure 2(b) did not receive the whole second beacon train. Upon reception of the first beacon from the third beacon train, this neighbor can realize that the second beacon train is lost and how many beacons are included in that beacon train through checking the beacon train number and comparing the sequence number of the currently received packet with the sequence number of the last received beacon. By identifying the number of lost beacon trains and the time interval between last received beacon train and complete reception of the current beacon train the receiver node can estimate the beaconing interval of the transmitter.

## 5. Simulation Model

This section presents the considered simulation parameters and simulation scenarios to evaluate and compare the performance of CNE, ANI-SB, and ANI-MB protocols. Furthermore, the last part of this section is dedicated to describe the utilized evaluation metrics and the main reason behind each measurement.

### 5.1. Simulation Setup

We used OMNeT++ framework to develop a simulation software. In order to provide an accurate wireless channel model and improve confidence on the validity of simulation results, we have developed a physical layer module based on the link model of [20] that considers path loss, multipath effect, transmission power variations, noise floor variations, and capture effect.  Table 1 presents the default simulation parameters of this paper. The radio parameters are chosen based on the characteristics of Mica2 motes with CC1000 radio. Furthermore, we have implemented the TinyOS's default CSMA MAC protocol in the simulation software. The initial and congestion backoff duration are 256 and 128, slots respectively. In all the experiments sensor nodes are deployed in a grid topology and we changed the spacing between nodes to produce different node densities.

The introduced initialization protocols in Section 4 are evaluated in the following scenarios.

- One-hop topology: in this scenario, all the nodes are within communication range of each other. We change the neighborhood size of nodes from 10 to 50 by varying the number of nodes from 10 to 50 in a grid topology. Since there is no hidden node collision in the one-hop topology, this scenario aims to focus on the effects of providing interleave beacon exchange pattern on the performance of the network initialization protocols.
- Multihop topology: we study the scalability of different network initialization protocols through this scenario. In fact, the main objective of these evaluations is to analyze the effects of network size variations on the performance of different methods. In contrast with the first scenario, in multihop scenario nodes cannot communicate with each other directly. Therefore, these experiments highlight the efficiency of different protocols to eliminate the effects of packet collisions due to the concurrent transmissions and hidden node problem on the performance of different protocols.

### 5.2. Performance Parameters

We have evaluated and compared the performance of CNE, ANI-SB, and ANI-MB protocols through following parameters.

*Number of Discovered Neighboring Nodes per Node*. This metric indicates the average neighborhood density that can be identified by different network initialization protocols. Performance evaluation through this metric reveals the capability of different protocols for discovering the exact number of neighboring nodes per node. 

*Link Estimation Errors*. This metric shows the difference between estimated link quality and actual data transmission quality of the identified links through measuring the average Root Mean Square Error (RMSE) of link quality estimations. In this measurement, the difference between actual data transmission quality of every link and its corresponding estimated value by CNE, ANI-SB, and ANI-MB protocols is squared and then averaged over the entire available links which have experienced at least one packet transmission. Finally, the square root of the calculated average value is taken. Actual data transmission quality of the links is calculated according to the distance between nodes as computed in [20].

*Network Initialization Period*. It demonstrates the duration of neighbor discovery and link quality estimation process. Accordingly, it presents the duration between transmission of the first beacon message and reception of the last beacon at the last node in the network. This comparison highlights the effects of adaptive and fixed beaconing rate adjustments on the network initialization period.

*Total Number of Packet Corruptions*. This metric expresses the total number of collisions during neighbor discovery and link quality estimation process. It is defined as the number of incoming packets at individual nodes that have resulted in a packet loss. This metric shows the ability of different methods to reduce the effects of packet collision on the accuracy of neighbor discovery and link quality estimations.

*Network Initialization Overhead Cost*. This metric reveals the overhead cost of running different network initialization protocols on multihop sensor networks in term of average percentage of energy consumed by individual nodes for network initialization.

## 6. Performance Analysis

In this section, we analyze and compare the performance of the presented network initialization protocols in Section 4 under one-hop and multihop scenarios. In all of the graphs, each result point shows the median of 10 simulation runs. Note that, for the CNE protocol, the beaconing rate is fixed to 1 beacon/second in all of the experiments [23, 29, 30]. Moreover, in CNE and ANI-SB, whenever a node wants to broadcast a beacon message, it adds the identity and number of received packets from its identified neighbors into a one beacon message. However, if the available space in the beacon message is not sufficient to include the related information of the entire identified neighbors, the sender node selects the entries through performing a round robin procedure [7, 30]. In contrast, in ANI-MB the sender node adds all of its neighborhood information into the multiple beacons and broadcasts them consecutively. Notice that number of beacons in all the graphs indicates the predefined number of times that each node should broadcast its neighborhood information. Moreover, in all the figures “*Y*” and “*k*” indicate the network and neighborhood size, respectively.

### 6.1. Performance Evaluation under First Simulation Scenario

In the following, we discuss about the effects of neighborhood density on the performance of network initialization protocols. Since in this scenario all the nodes are in the communication range of each other, we only evaluate performance of different protocols in terms of link quality estimation errors and network initialization period while all of the introduced parameters in Section 5.2. are evaluated under multihop scenario.

#### 6.1.1. Link Estimation Errors

**Figure 3:**
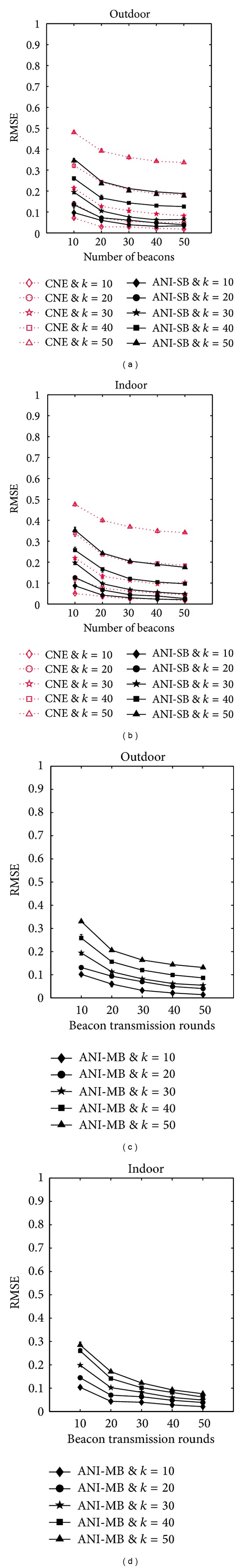
Link quality estimation errors by ANI-SB, ANI-MB, and CNE protocols in one-hop topology networks deployed in outdoor and indoor environments.

Figures 3(a) and 3(b) show the RMSE of the provided estimations by ANI-SB and CNE protocols in outdoor and indoor environments, respectively. Since in these experiments all the nodes are in the transmission range of each other, environmental setting has not affected the performance of different protocols and each protocol provides similar link estimation errors in outdoor and indoor environments. As it can be observed from these figures, ANI-SB protocol improves the accuracy of link estimations up to 100% and 50% compared to the CNE protocol in high and medium densities, respectively. According to these figures by increasing the number of beacons from 10 to 30, CNE protocol slightly improves errors of link quality estimations by 30% in outdoor and indoor environments. Nevertheless, further increases in the number of beacon messages cannot enhance the precision of the achieved link quality estimations, while the ANI-SB protocol improves accuracy of the link estimations in high neighborhood densities (i.e., 50 and 40 neighbors [52, 53]) up to 47%, by increasing the number of beacons from 10 to 50. These behaviors can be justified as follows: in order to control packet collisions during broadcasts, existing CSMA-based MAC protocols use carrier sensing mechanism to allow nodes to detect ongoing transmissions. By using carrier sensing mechanism, whenever a node wants to broadcast a beacon message it chooses a random backoff time *t* from the specified contention window (0, CW) and waits for *t* slots before attempting for a broadcast. As nodes select a random transmission slot to perform broadcasts, packet collisions due to the concurrent transmissions are still possible. Although in carrier sensing mechanism, if a node with a new beacon message to transmit senses the channel is busy, it defers its transmission by performing a random backoff time; still there is a high probability of packet collision right after a positive carrier sensing. Therefore, identifying the beaconing rate of the nodes without considering the number of concurrent interfering transmitters cannot provide-collision free broadcasts in different network densities. Since, in the proposed ANI-SB protocol, each node adapts its beaconing rate according to the beaconing rate of its neighboring nodes, network initialization through this protocol takes more time to rectify the beaconing rate of individual nodes by transmitting more beacons. However, broadcasting a lot of beacon messages in CNE protocol cannot help to reduce the errors in the link estimations, while it also intensifies packet collisions due to the concurrent transmissions. In fact, as in CNE protocol all the nodes select their transmission time from a fixed interval independently; so there is a high probability for overlapping broadcasts of several nodes, which causes collision in the receiver nodes. Figures 3(a) and 3(b) also show the estimation errors of both protocols degrade as the number of neighboring nodes falls. The reason is that reducing the neighborhood size in one-hop topology decreases the number of concurrent transmissions which will cause packet collisions.

Figures 3(c) and 3(d) depict the effects of multiple beaconing on the performance of the adaptive protocol in outdoor and indoor environments, respectively. As can be seen from these figures, multiple-beaconing technique enhances the accuracy of the estimations about 35% compared to the ANI-SB protocol in the high density networks. However, it can not cause any improvement in the accuracy of link estimations for neighborhood size of 10. The main reason behind this behavior is that with neighborhood size of 10, the available space of a single beacon is enough to share the neighborhood information of nodes while the available space in a single beacon is insufficient to cover the entire neighborhood information of individual nodes in high density networks. Consequently, in high network densities each broadcast can only share the preserved information related to a set of nodes. In contrast, by broadcasting the whole neighborhood information through transmitting multiple tandem beacons, nodes are able to share their whole neighborhood information during all the beacon transmission rounds. Accordingly, all the neighboring nodes of individual nodes can update the PRR of their outgoing links based on the last values. Furthermore, ANI-MB protocol enables each node to share its entire neighborhood information several times through broadcasting beacon trains. Thus packet collisions have less impact on the accuracy of the estimations compared to the ANI-SB mechanism. On the other hand, since in the ANI-SB protocol nodes select the entries of individual beacons by a round robin procedure, every single packet collision can increase the link estimation errors.

#### 6.1.2. Network Initialization Period

**Figure 4:**
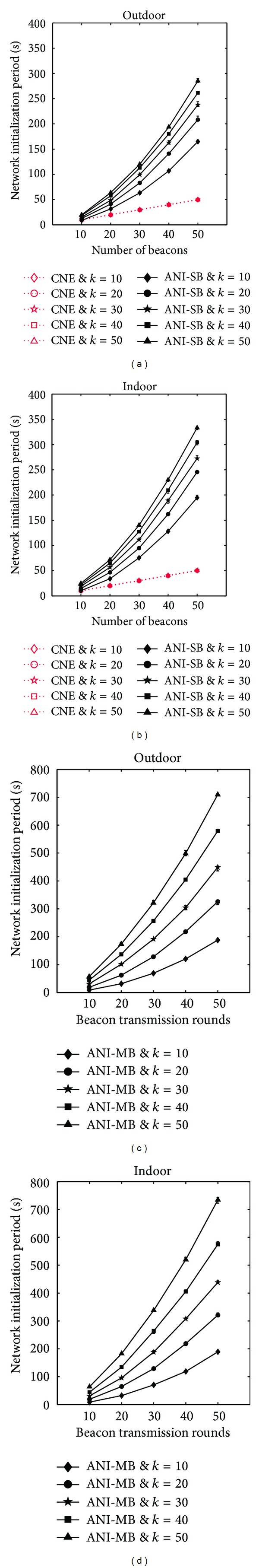
Neighbor discovery and link quality estimation period by ANI-SB, ANI-MB and CNE protocols in one-hop topology networks deployed in outdoor and indoor environments.

The network initialization period through different protocols in outdoor and indoor environments are shown in Figures 4(a) and 4(b). According to these figures, duration of the network initialization process by CNE protocol is linearly related to the number of broadcasted beacon messages. Since all the nodes have a same beaconing interval, increasing the number of beacons elevates the network initialization duration. Same as CNE protocol, as the neighborhood size of the nodes scales up, the network initialization period through ANI-SB protocol also increases. As it is perceived ANI-SB protocol causes higher network initialization duration compared to CNE protocol. The reason is that, in ANI-SB protocol, every node increases its waiting period before broadcasting a new beacon message by identifying new neighboring nodes, while in CNE protocol each node broadcasts a new beacon message every one second without considering its neighborhood density. Through performing ANI-SB protocol, as the neighbor discovery proceeds, each node identifies new neighbors and updates its beacon transmission interval based on the beaconing interval of its neighbors. Therefore, increasing the neighborhood size of network nodes, elevates the beaconing interval of individual nodes which in turn intensifies the network initialization duration. Based on the achieved results the maximum efficiency of employing ANI-SB protocol in reducing the link quality estimation errors under high neighborhood densities (i.e., 50 neighbors) can be achieved by transmitting up to 30 beacon messages and maximum network initialization duration of 2 minutes.

The effect of employing multiple-beaconing technique in the adaptive protocol on the network initialization period is illustrated in Figures 4(c) and 4(d) for outdoor and indoor environments, respectively. Based on these figures ANI-MB protocol intensifies the network initialization duration compared to the ANI-SB protocol in high network densities. This behavior can be justified by the fact that, in low network densities (e.g., 10 neighbors), the neighborhood information of each node can be shared through a single beacon. Nevertheless, as the number of neighbors increases, the network initialization period also grows according to the neighborhood size. For instance, when each node shares its neighborhood information 30 times in the neighborhood size of 50, ANI-MB protocol elevates the network initialization duration about 100% compared to ANI-SB protocol. While, employing this technique with a same setting in neighborhood size of 20, increases the network initialization duration up to 43% compared to the ANI-SB protocol.

**Figure 5:**
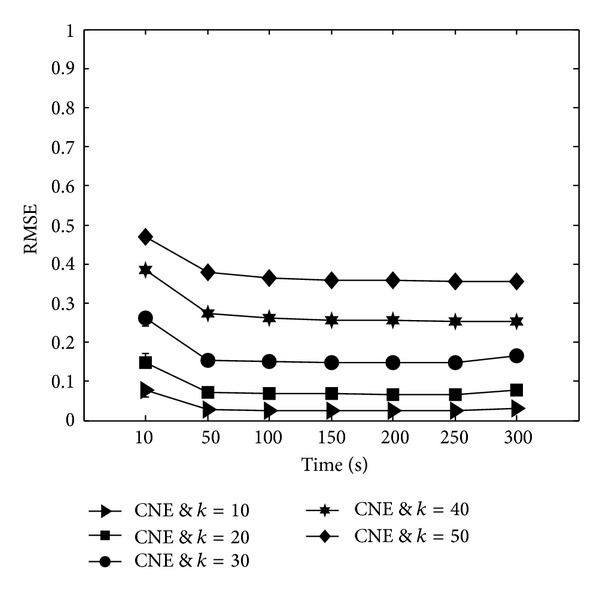
Variations of estimation errors in the CNE protocol with 1 beacon/second beaconing rate versus network initialization in one-hop topology networks deployed in outdoor environment.

Moreover, we analyze the effects of network initialization period on the accuracy of link estimations in the CNE protocol to show the cost of deterministic network initialization duration feature of this approach in term of link estimation errors. As mentioned before, in the CNE protocol all the nodes have a same beaconing interval which should be determined before network initialization based on the user-defined discovery length and number of beacon transmissions. In order to highlight the effects of beaconing rate adjustment on the accuracy of link estimations, in this performance study the beaconing rate of all the nodes is fixed to 1 beacon/second while the network initialization period increases from 10 seconds to 300 seconds. As can be observed from Figure 5, just extending the network initialization period through broadcasting more beacon messages without defining a proper beaconing interval for individual nodes does not necessarily reduce the link quality estimation errors. On the other hand, this observation justifies that beaconing rate of network nodes directly affects the accuracy of the link estimations. The general conclusion of these simulation studies is that appropriate beaconing interval adjustment can help to improve the accuracy of the initial link quality estimations.

### 6.2. Performance Evaluation under Second Simulation Scenario

In the second scenario, we focus on the effects of multihop network topology with medium and high neighborhood densities (i.e., 20 and 50 neighbors [53–55]) on the performance of network initialization protocols in terms of number of discovered neighbors per node, link estimation errors, network initialization period, number of packet corruptions, and network initialization overhead cost. Accordingly, in each configuration we changed the network size (i.e., 100, 200, 400, and 600 nodes) while the network density maintained at a fixed size (i.e., 20 or 50 neighbors).

#### 6.2.1. Number of Discovered Neighboring Nodes per Node

**Figure 6:**
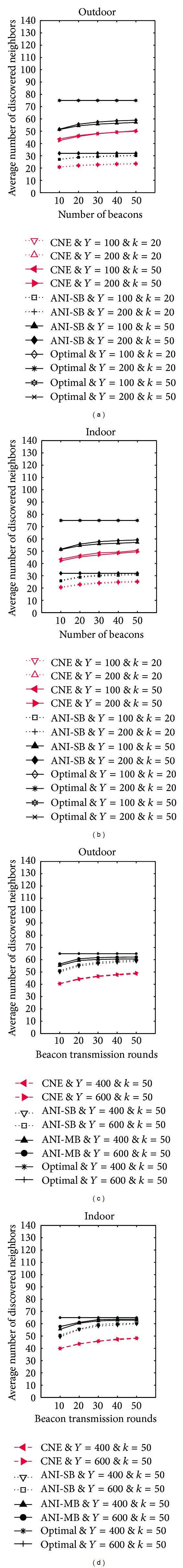
Average number of discovered neighbors by ANI-SB, ANI-MB, and CNE protocols in multihop topology networks deployed in outdoor and indoor environments.

Figures 6(a) and 6(b) show the number of discovered neighbors by ANI-SB and CNE protocols in multihop networks which are deployed in outdoor and indoor environments. Although the results in each figure are demonstrated based on different neighborhood densities in networks with dissimilar sizes, the identified neighborhood size in these figures cannot indicate the exact number of potential neighbors per node. This is due to the fact that the neighborhood size measurements for performance evaluations are computed as the average number of neighbors per node for which their corresponding link quality is more than 10%. Nevertheless, due to the high variations of link quality in wireless networks, this method results in rough estimations on the actual neighborhood density. Accordingly, in this study an *optimal protocol* is used as a baseline to compare the efficiency of different protocols to discover maximum number of neighboring nodes. In the optimal protocol every node broadcasts 100 beacon messages and considers all the nodes from which it has received at least one beacon message as its potential neighboring nodes.

Since the distance between nodes in each network is adjusted in such a way that the neighborhood density remains constant in both environments (i.e., 20 and 50 neighbors), so each protocol exhibits almost a same behavior in both environments. According to Figures 6(a) and 6(b), the average number of discovered neighbors per node through the ANI-SB is about 30% higher than the CNE protocol. For instance with 10 and 50 beacon transmissions ANI-SB protocol detects about 9 and 10 neighbors more than CNE protocol, respectively. The main reason is that in high neighborhood densities adjusting the beaconing rate of the nodes based on the beaconing rate of their neighbors reduces the probability of packet collisions which in turn increases the chance of receiving at least one beacon message from potential neighboring nodes.

Figures 6(c) and 6(d) represent that employing ANI-MB elevates the number of discovered nodes by every node about 15% and 43% compared to the ANI-SB and CNE protocols in large-scale sensor networks which are deployed in outdoor and indoor environments, respectively. According to these figures, ANI-MB protocol discovers the same number of neighboring nodes as the optimal protocol when it performs 30 beacon transmission rounds at individual nodes. This high performance of the ANI-MB protocol is the result of transmitting multiple beacons in each beacon transmission round. In fact, transmitting the related information of a single node at each round increases the chance of receiving at least one of the transmitted packets by its neighbors.

#### 6.2.2. Link Estimation Errors

**Figure 7:**
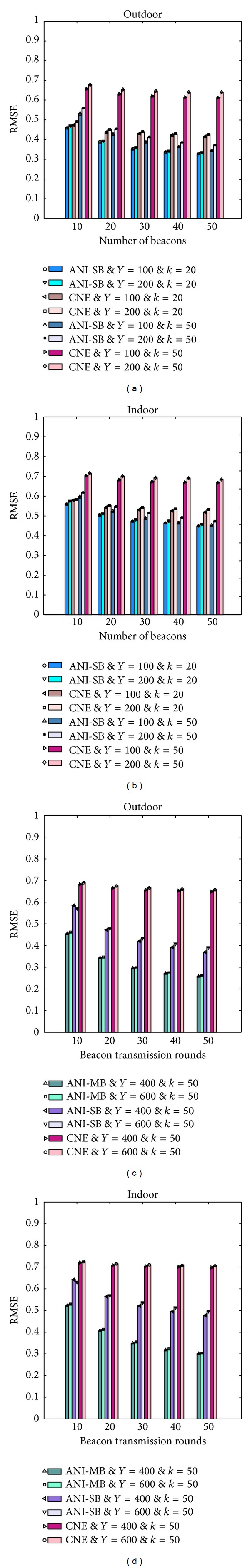
Link quality estimation errors by ANI-SB, ANI-SB, and CNE protocols in multihop topology networks deployed in outdoor and indoor environments.

Figures 7(a) and 7(b) demonstrate accuracy of the provided link estimations by ANI-SB and CNE protocols in outdoor and indoor environment with various network sizes. Moreover, these figures also show the effects of changes in the network density of the multihop topology on the link estimation errors. According to these figures link estimation errors through different protocols in indoor environment are higher than outdoor environment. This behavior is due to higher multipath channel variations of the indoor environment compared to the outdoor environment. In fact, high multipath channel variations in the indoor environment increase the size of transitional region which in turn raises the number of intermediate-quality links in the network. Due to the high variations of intermediate quality links, increasing the number of intermediate-quality links elevates the link estimation errors.

It can be inferred from these figures that ANI-SB improves the accuracy of link estimations up to 75% and 32% compared to CNE protocol in neighborhood density of 50 and 20 nodes, respectively. The higher performance of the ANI-SB is the result of adjusting beaconing interval of the nodes during network initialization period. In fact, adjusting the waiting period of nodes based on the beaconing rate of their neighbors before each broadcast reduces the probability of link quality estimation errors due to collisions. The network size variations in the multihop topology do not significantly affect the accuracy of the provided link estimations by both protocols. This observation suggests that, in contrast with network density, network size of the multihop topology does not strongly influence the accuracy of initial link assessments. Another observation that can be drawn from this experiment is that increasing the number of beacons cannot help to improve the accuracy of link estimations in CNE protocol. The reason is that beaconing rate of all the nodes in this protocol is adjusted to 1 beacon/second, regardless of the network density and number of beacons [23, 29, 30]. On the other hand, increasing the number of beacons without adjusting the beaconing rate of the nodes only elevates the amount of packet collisions, which directly affects the accuracy of link estimations. This is the shortcoming of the CNE protocol as it determines the beaconing rate of the nodes before network deployment. In contrast, by increasing beacon transmissions in ANI-SB protocol, network nodes have more time to adjust their beaconing intervals. As a consequence, by increasing the number of beacon messages the errors in the provided link quality estimations by ANI-SB protocol are reduced by 40% and 32% for neighborhood density of 50 and 20 nodes, respectively. Furthermore, Figures 7(a) and 7(b) indicate that increasing the number of beacon transmissions declines the effects of neighborhood size variations on the errors of provided link estimations by ANI-SB protocol. In contrast, according to Figures 7(a) and 7(b), as the neighborhood density of a network with a fixed number of nodes scales up, the estimation errors of the CNE protocol raise by 55%. This observation demonstrates the scalability of the proposed adaptive protocol, which enables this protocol to work efficiently under different network topologies. This means that adaptive protocol can provide accurate results without being influenced by the network size or neighborhood density of the nodes.

By comparing these results with the achieved results in one-hop scenario, it can be observed that multihop topology increases the errors in the achieved estimations through different protocols about 70%. This observation can be justified by the fact that multihop topology causes more collisions than the one-hop topology because of hidden terminal problem. As a consequence, the effects of packet collisions on link quality estimation errors in the multihop topology always higher than the one-hop topology. In the one-hop topology, all the nodes are able to communicate with each other directly, and there is no hidden node, so concurrent transmissions of the neighboring nodes in a same slot are the main cause of collisions, which result in link quality estimation errors. In contrast, in the multihop topology, radio range of every node does not cover the entire network, which arises hidden terminal problem and complicates the collision-free transmissions [47]. In fact, packet collision due to hidden terminal problem can take place, if at least two nodes transmit their packets concurrently to a same node which is a common neighbor for these transmitters. Therefore, there are two sources of packet collisions in a multihop network: two neighboring nodes, which are visible to each other, randomly select a same transmission slot or hidden terminal problem.

Figures 7(c) and 7(d) show the effects of employing multiple-beaconing technique through ANI-MB protocol on the accuracy of the provided link estimations compared to the ANI-SB, and CNE protocol in large-scale networks deployed in outdoor and indoor environments, respectively. In general, still link estimation errors in the indoor environment are higher than the outdoor environment due to its higher multipath channel variations. As can be seen from these figures by increasing the number of beacon trains up to 30, ANI-SB and ANI-MB protocols decline the errors of link estimations about 63% and 50% in outdoor and indoor environments, respectively, while the RMSE of link estimation through CNE protocol remains constant in both environments. Furthermore according to Figure 7(c), ANI-MB reduces the link estimation errors up to 50% and 142% compared to the ANI-SB and CNE protocols, respectively. The main reason behind this behavior is that the available space in a single beacon is not sufficient to include the whole neighborhood information of the nodes in large-scale networks with high density. Therefore, through ANI-MB protocol nodes are able to share their neighborhood information through consecutive beacons during all the beacon transmission rounds. By this way all the neighboring nodes of every node can update the PRR of their outgoing links based on the last updates on the number of successfully transmitted beacons. Moreover, as in ANI-MB every node broadcasts its neighborhood information at each beacon transmission round, packet collisions have less impact on the accuracy of the estimations compared to the ANI-SB and CNE protocols.

#### 6.2.3. Network Initialization Period

**Figure 8:**
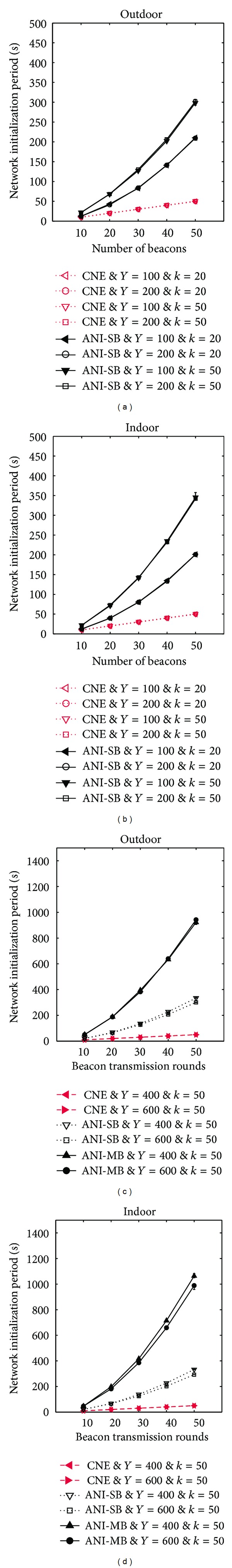
Neighbor discovery and link quality estimation period by ANI-SB, ANI-MB, and CNE protocols in multihop topology networks deployed in outdoor and indoor environments.

**Figure 9:**
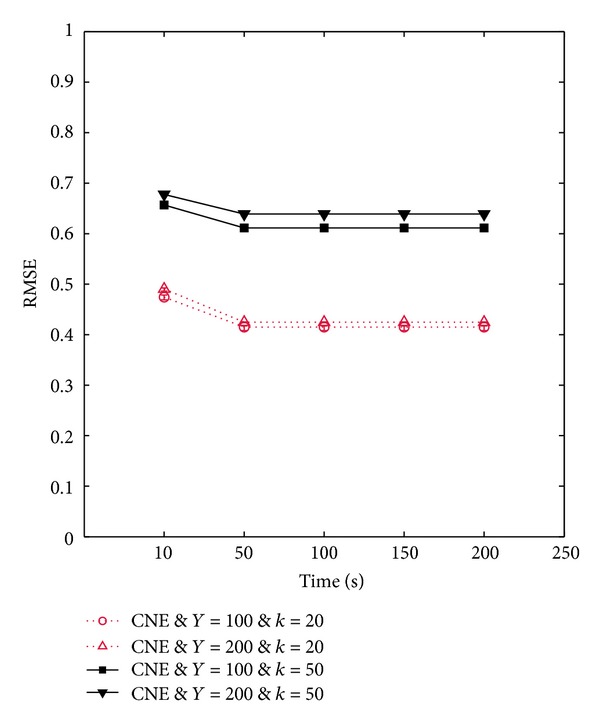
Variations of the link estimation errors in the CNE protocol with 1 beacon/second beaconing rate versus network initialization duration in multihop topology networks deployed in outdoor environment.

The required time for initializing a multihop network through ANI-SB and CNE protocols in outdoor and indoor environments is illustrated in Figures 8(a) and 8(b), respectively. By comparing these figures, it can be observed that the network initialization period through each protocol is similar in both environments. According to Figure 8, CNE protocol can provide deterministic network initialization duration regardless of the network size and neighborhood density. Based on this figure, the network initialization duration in CNE is only related to the beaconing rate of the nodes and number of beacon messages which are defined by the network administrator before network initialization. As can be observed from Figure 9, increasing the network initialization period through transmitting more beacon messages without selecting appropriate beaconing interval does not necessarily improve the accuracy of the link estimations. Note that although CNE protocol can provide deterministic network initialization duration, it requires correct information regarding the neighborhood density of individual nodes before network initialization to provide accurate link quality estimations. In fact, the channel load which may cause a high packet collision rate at the saturated levels is a function of nodes beaconing rate and network initialization period. Therefore, the accuracy of the link estimations and neighbor discovery highly depends on the selected beaconing interval and link quality estimation period. In contrast, as the ANI-SB protocol adjusts the beaconing intervals of the nodes according to their neighborhood density, the duration of network initialization through this protocol is related to the network density. As can be seen from Figure 8, increasing the neighborhood size of the nodes elevates the network initialization duration, while it is not affected by the network size variations. As a consequence, the proposed protocol can efficiently reduce link quality estimation errors due to the packet collisions in the cases where the network topology is unknown (e.g., when the sensor nodes are dropped from a helicopter in the area of interest).

Figures 8(c) and 8(d) demonstrate the delay of neighbor discovery and link quality estimation process through CNE, ANI-SB, and ANI-MB protocols in large-scale networks with neighborhood size of 50 nodes in outdoor and indoor environments, respectively. By comparing Figure 8(c) with Figure 8(d), it can be seen that each protocol causes almost a same network initialization period under different network sizes and environmental settings. As it is expected, ANI-MB protocol increases the delay of network initialization compared to the ANI-SB protocol. As it has been shown in Figure 7, ANI-MB can reduce the errors of link estimation through transmitting up to 30 beacon trains and further increases in the number of beacon trains cannot help to improve the accuracy of link estimations. Therefore, ANI-MB protocol reduces the errors of link estimations by 50% compared to the ANI-SB protocol while it raises the network initialization period by 100%.

The general conclusion from these simulation results is that, the beaconing rate of individual nodes should be tuned adequately in order to improve accuracy of neighbor discovery and link estimation process through increasing the network initialization period. According to the simulation results ANI-SB and ANI-MB protocols can provide high accuracy without requiring any parameter adjustment by the system engineer before network deployment. Therefore, the proposed protocols are especially useful when the network topology is unknown.

#### 6.2.4. Number of Packet Corruptions

**Figure 10:**
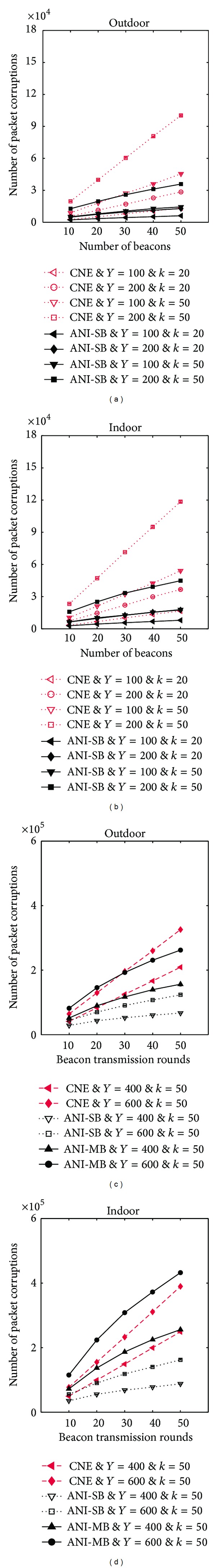
Number of packet corruptions by ANI-SB, ANI-MB, and CNE in multihop topology networks deployed in outdoor and indoor environments.

Packet corruption analysis examines the capability of ANI-SB, ANI-MB, and CNE protocols to eliminate the effects of packet collisions on the link estimations. A packet corruption can happen in the developed simulation software if during the reception of a beacon message another broadcasted message is being heard by the receiver. The environmental effects on the number of packet corruptions caused by employing different protocols are highlighted in Figures 10(a) and 10(b). With a given number of beacon transmissions, the number of packet corruptions in indoor environment is about 25% higher than the outdoor environment. This is due to the lower path loss exponent of the indoor environment which increases the number of high-quality interfering signals compared to the outdoor environment. Since a large number of packet receptions in the indoor environment are affected by the high-quality interfering signals, the number of packet corruptions in this situation is higher than the outdoor environment.

Figures 10(a) and 10(b) indicate the collision avoidance capability of the ANI-SB protocol is significantly better compared to the CNE protocol. In fact the number of packet corruptions in the CNE protocol is about 260% and 120% higher than the ANI-SB protocol in neighborhood density of 50 and 20 nodes. As can be observed in Figures 10(a) and 10(b), as the number of beacon messages increases, the number of beacon corruptions in CNE protocol rises about 400% for the neighborhood size of 50 while in the ANI-SB protocol, by raising the number of beacon messages, the incremental trend of packet corruptions is not significant. These observations signify the efficiency of the proposed protocol in adjusting the beaconing interval of individual nodes according to their neighborhood density which eliminates the effects of packet collisions on the link quality estimations. In addition, these figures show that the collision avoidance capability of both protocols is influenced by variations of the network size and neighborhood density. According to Figure 10, elevating either network or neighborhood size extremely increases the total number of packet corruptions in the CNE protocol. The reason is that performing packet broadcast by all the nodes in high network densities without controlling the broadcast rate of individual nodes causes a high channel load, which in turn increases packet collisions. Still, the proposed protocol demonstrates that raising the neighborhood size causes small variations in the number of packet corruptions compared to the CNE protocol. This behavior is expected, because in the proposed protocol every node waits for receiving a beacon message from all of its identified neighboring nodes before it broadcasts a new beacon message at each round of beacon transmission. This waiting time adjustment at individual nodes before each broadcast significantly reduces the probability of packet collisions due to the concurrent transmissions or hidden terminal problem. The same behavior also holds for the effects of increasing beacon messages on the total number of packet corruptions during the network initialization phase. As it can be seen from Figure 10, by increasing the number of beacon messages in ANI-SB protocol, the slope of the curve for different neighborhood size slightly increases while Figure 10 shows the number of packet corruptions during network initialization through the CNE protocol sharply increases as the number of beacon messages raises. This behavior confirms the inappropriateness of the increasing number of beacons in the CNE protocol for improving the accuracy of link estimations, without making any change in the beaconing rate of the nodes when the network size and neighborhood density scale up.

The total number of packet collisions caused during initialization of large-scale dense wireless sensor networks in outdoor and indoor environments through ANI-MB, ANI-SB, and CNE protocols are depicted in Figures 10(c) and 10(d). Since the ANI-MB protocol increases the number of beacon transmissions in the network, it elevates the number of packet collisions about 100% compared to the ANI-SB protocol. Furthermore, these figures show that increasing the network size raises the packet collisions by 100% in CNE and ANI-MB protocols and about 40% in ANI-SB protocol.

#### 6.2.5. Network Initialization Overhead Cost

**Figure 11:**
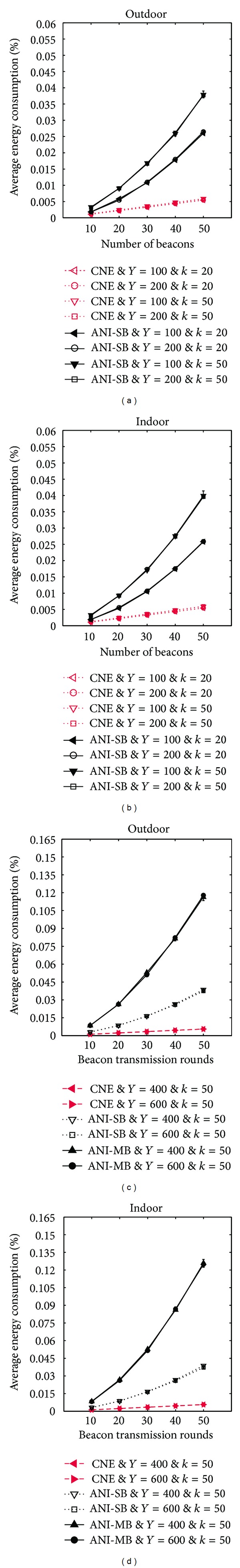
Overhead cost of running ANI-SB, ANI-MB, and CNE protocols in multihop topology networks deployed in outdoor and indoor environments.

This section analyzes the overhead cost of running ANI-SB, ANI-MB, and CNE protocols in multihop sensor networks in term of average energy consumption at individual nodes for network initialization. Figures 11(a) and 11(b) demonstrate the average consumed energy (in percentage) of the total battery capacity of a sensor node through individual protocols is similar for outdoor and indoor environments. As it is expected, the average energy consumption of nodes in the CNE protocol is lower than the ANI-SB protocol. The reason is that, in the ANI-SB protocol each node should wait for its one-hop neighboring nodes to broadcast their beacons before broadcasting a new beacon message. As a result, the average idle listening duration through the ANI-SB protocol is higher than the CNE protocol. Although the energy consumption in receive and transmit modes is different, the energy consumption of the idle listening mode is almost the same as that of receive mode. Therefore, higher idle listening duration in the ANI-SB protocol results in more energy consumption through this protocol compared to the CNE protocol. According to Figures 11(a) and 11(b), as the neighborhood size of nodes scales up the average energy consumption, percentage of nodes also increases. This can be justified by the fact that increasing the neighborhood density elevates the beaconing interval of individual nodes, which in turn raises the average idle listening duration of the nodes. In contrast, neighborhood size variations do not affect the energy consumption of the individual nodes for initializing a multihop network through the CNE protocol. The reason is that the beaconing rate of all the nodes is fixed to 1 beacon/second in all the configurations, which results in a constant idle listening duration. Furthermore, network size variations do not affect the energy consumption of the nodes in both protocols. Despite the higher energy consumption of the ANI-SB protocol than CNE protocol, still its operation consumes about 0.0025% and 0.038% of the total battery capacity of a node with 2500 mAh and 3 V battery, when every node in a multihop network with high neighborhood density broadcasts 10 and 50 beacon messages, respectively. Moreover, according to Figure 7 transmitting more than 30 beacons in ANI-SB protocol cannot help to provide further improvements in the accuracy of link estimations. So, the maximum energy consumption of the proposed protocol at a single node is about 0.015% and 0.01% of the total battery capacity of a node in the networks with neighborhood density of 50 and 20 nodes, respectively. Therefore, cost of performing accurate neighbor discovery and link quality measurement in term of nodes energy consumption is not significant. Figures 11(c) and 11(d) illustrate the consumed energy of the total battery capacity of a sensor node through running ANI-SB, ANI-MB, and CNE protocols in the large-scale wireless sensor networks which are deployed in indoor and outdoor environments. The trend of the curves in Figure 11(c) is similar to the trend of their pairs in Figure 11(d). Since in this experiment the network size is grown-up to 400 and 600 nodes, the overhead cost of all the protocols is increased compared to the previous experiment. Still the proposed ANI-SB and ANI-MB protocols reduce the errors of link estimations about 50% and 142% compared to the CNE protocol through consuming about 0.02% and 0.05% of the total battery capacity of a sensor node (with 30 beacon transmission rounds).

## 7. Conclusion

In this paper, we have proposed two adaptive network initialization protocols to demonstrate the efficiency of adaptive protocols in comparison with nonadaptive protocols to provide accurate information about availability and quality of the communication links at the network initialization phase. Furthermore, we have performed an extensive simulation study on the proposed adaptive protocols and the existing nonadaptive network initialization protocols through OMNeT++ simulation framework. First, we have evaluated the accuracy of link estimations and network initialization period through different protocols independent of the impacts of high packet collisions which cause by multihop nature of low-power wireless networks. The results show that since upon network deployment nodes do not have any information regarding their vicinity, adapting the beaconing rate of individual nodes based on the beaconing rate of their neighbors during network initialization period highly reduces the errors of primary link estimations. Nevertheless, eliminating the packet collisions at the initialization phase through increasing the beacon transmission interval of the nodes elevates the network initialization period. Next, it has demonstrated how different protocols can reduce the effects of extensive packet collisions in the multihop topologies on the link quality estimation errors in order to provide accurate information regarding the available links and their quality prior to topology formation. The achieved results from multihop scenario confirm the application independence of the adaptive protocols which can provide easy adaptability of a wireless sensor network to the new applications. Based on the simulation results, the accuracy of the provided information by the nonadaptive protocols highly depends on tuning the beaconing rate of nodes before network topology construction. However, in most of the applications it is hard to define an appropriate beaconing rate which results in collision-free beacon exchange between nodes without prior knowledge about network topology. Finally, we have examined the overhead cost of different protocols in term of average energy consumption at individual nodes for initializing a multihop network. The results indicate that adaptive protocols can provide efficient network initialization through consuming a small percentage of available battery capacity of a single sensor node.

In general this study shows the proposed adaptive protocols can provide more accurate information regarding the availability of wireless links along with their data transmission quality upon network deployment compared to the existing nonadaptive protocols, yet they do not satisfy at best all the performance parameters. In fact, supporting reliable neighbor discovery and link quality estimation regardless of network topology and neighborhood density has a cost in term of network initialization period. Therefore, it is significant to provide an efficient tradeoff between accuracy of the link estimations and network initialization period according to the performance demands of different applications.

## Figures and Tables

**Table 1:** Simulation parameters.

Radio	
Transmission power (dBm)	0
Average noise power (dBm)	−106
Switch to TX/RX (*μ*s)	250
Radio sampling (*μ*s)	350
Evaluate radio sample (*μ*s)	100
Modulation	NC-FSK
Encoding	Manchester
Baud rate	38400
Radio speed after encoding (bits/second)	19200
Transmission power variations	1.1
Noise floor variations	0.9
Reference distance (*d*_0_) (m)	1
PL (*d*_0_) (dB)	55
Current consumption in transmit mode (mA)	16.5
Current consumption in receive mode (mA)	9.6
Current consumption in idle mode (mA)	9.6
Current consumption in sleep mode (mA)	0.0000002
Battery capacity (mAh)	2500

Environment	

Path loss exponent (indoor/outdoor)	3.3/4.7
Multipath channel variations (indoor/outdoor)	5.5/3.2

MAC	

Initial backoff (slots)	256
Congestion backoff (slots)	128
Carrier sensing threshold	CCA

Packet format	

Preamble size (byte)	10
MAC header size (byte)	5
Payload size (byte)	29
CRC size (byte)	2

## References

[1] Yick J, Mukherjee B, Ghosal D (2008). Wireless sensor network survey. *Computer Networks*.

[2] Radi M, Dezfouli B, Bakar KA, Razak SA, Lee M (2013). Network initialization in low-power wireless networks: a comprehensive study. *The Computer Journal*.

[3] Galluzzi V, Herman T (2012). Survey: discovery in wireless sensor networks. *International Journal of Distributed Sensor Networks*.

[4] Abdesslem FB, Iannone L, de Amorim M, Obraczka K, Solis I, Fdida S An abstraction layer for neighborhood discovery and cross-layer metrics.

[5] Bachir A, Dohler M, Watteyne T, Leung KK (2010). MAC essentials for wireless sensor networks. *IEEE Communications Surveys and Tutorials*.

[6] Rhee I, Warrier A, Min J, Xu L DRAND: distributed randomized TDMA scheduling for wireless ad-hoc networks.

[7] Colesanti U, Santini S (2011). The collection tree protocol for the castalia wireless sensor networks simulator.

[8] Akkaya K, Younis M (2005). A survey on routing protocols for wireless sensor networks. *Ad Hoc Networks*.

[9] Radi M, Dezfouli B, Bakar K, Lee M (2012). Multipath routing in wireless sensor networks: survey and research challenges. *Sensors*.

[10] Li M, Baijian Y A survey on topology issues in wireless sensor network.

[11] Dezfouli B, Radi M, Razak SA (2010). A cross-layer approach for minimizing interference and latency of medium access in wireless sensor networks. *International Journal of Computer Networks and Communications*.

[12] Dezfouli B, Radi M, Nematbakhsh MA, Razak SA A medium access control protocol with adaptive parent selection mechanism for large-scale sensor networks.

[13] Rhee I, Warrier A, Aia M, Min J, Sichitiu ML (2008). Z-MAC: a hybrid MAC for wireless sensor networks. *IEEE/ACM Transactions on Networking*.

[14] Moeller S, Sridharan A, Krishnamachari B, Gnawali O Routing without routes: the backpressure collection protocol.

[15] Baccour N, Mottola L, Niga MZ (2012). Radio link quality estimation in wireless sensor networks: a survey. *ACM Transactions on Sensor Networks*.

[16] Srinivasan K, Dutta P, Tavakoli A, Levis P (2010). An empirical study of low-power wireless. *ACM Transactions on Sensor Networks*.

[17] Zhao J, Govindan R Understanding packet delivery performance in dense wireless sensor networks.

[18] Srinivasan K, Dutta P, Tavakoli A, Levis P Understanding the causes of packet delivery success and failure in dense wireless sensor networks.

[19] Lal D, Manjeshwar A, Herrmann F, Uysal-Biyikoglu E, Keshavarzian A Measurement and characterization of link quality metrics in energy constrained wireless sensor networks.

[20] Zamalloa MZ, Krishnamachari B (2007). An analysis of unreliability and asymmetry in low-power wireless links. *ACM Transactions on Sensor Networks*.

[21] Radi M, Dezfouli B, Razak SA, Bakar KA LIEMRO: a Low-Interference energy-efficient multipath routing protocol for improving QoS in event-based wireless sensor networks.

[22] Radi M, Dezfouli B, Bakar KA, Razak SA, Nematbakhsh MA (2011). Interference-aware multipath routing protocol for QoS improvement in event-driven wireless sensor networks. *Tsinghua Science and Technology*.

[23] Baccour N, Koubaa A, Jamaa MB, Youssef H, Zuniga M, Alves M A comparative simulation study of link quality estimators in wireless sensor networks.

[24] Vlavianos A, Law LK, Broustis I, Krishnamurthy SV, Faloutsos M Assessing link quality in IEEE 802.11 wireless networks: which is the right metric?.

[25] Gnawali O, Jamieson K, Levis P, Fonseca R Four-bit wireless link estimation.

[26] Cerpa A, Wong JL, Potkonjak M, Estrin D Temporal properties of low power wireless links: modeling and implications on multi-hop routing.

[27] Gnawali O, Fonseca R, Jamieson K, Moss D, Levis P Collection tree protocol.

[28] Keshavarzian A, Uysal-Biyikoglu E, Herrmann F, Manjeshwar A Energy-efficient link assessment in wireless sensor networks.

[29] Meier A, Weise M, Beutel J NoSE: neighbor search and link estimation for a fast and energy efficient initialization of wsns.

[30] Couto D, Aguayo D, Bicket J, Morris R (2005). A high-throughput path metric for multi-hop wireless routing. *Wireless Networks*.

[31] McGlynn MJ, Borbash SA Birthday protocols for low energy deployment and flexible neighbor discovery in ad hoc wireless networks.

[32] Borbash SA, Ephremides A, McGlynn MJ (2007). An asynchronous neighbor discovery algorithm for wireless sensor networks. *Ad Hoc Networks*.

[33] Tseng YC, Hsu CS, Hsieh TY (2003). Power-saving protocols for IEEE 802.11-based multi-hop ad hoc networks. *Computer Networks*.

[34] Dutta P, Culler D Practical asynchronous neighbor discovery and rendezvous for mobile sensing applications categories and subject descriptors.

[35] Ye W, Heidemann J, Estrin D (2004). Medium access control with coordinated adaptive sleeping for wireless sensor networks. *IEEE/ACM Transactions on Networking*.

[36] Lu G, Krishnamachari B, Raghavendra CS Performance evaluation of the IEEE 802.15.4 MAC for low-rate low-power wireless networks.

[37] Kohvakka M, Suhonen J, Kuorilehto M, Kaseva V, Hännikäinen M, Hämäläinen TD (2009). Energy-efficient neighbor discovery protocol for mobile wireless sensor networks. *Ad Hoc Networks*.

[38] Javaid N, Javaid A, Khan IA, Djouani K Performance study of ETX based wireless routing metrics.

[39] Zuniga M, Krishnamachari B Analyzing the transitional region in low power wireless links.

[40] Ganesan D, Krishnamachari B, Woo A, Culler D (2002). Complex behavior at scale: an experimental study of low-power wireless sensor networks.

[41] García-Hernández CF, Ibargüengoytia-González PH, García-Hernández J, Pérez-Díaz JA (2007). Wireless sensor networks and applications: a survey. *International Journal of Computer Science and Network Security*.

[42] Gilbert EEPK, Baskaran K, Blessing EBE (2012). Research issues in wireless sensor network applications: a survey. *International Journal of Information and Electronics Engineering*.

[43] Kandhalu A, Lakshmanan K, Rajkumar R U-connect: a low-latency energy-efficient asynchronous neighbor discovery protocol.

[44] Hamida EB, Chelius G, Busson A, Fleury E (2008). Neighbor discovery in multi-hop wireless networks: evaluation and dimensioning with interference considerations. *Discrete Mathematics and Theoretical Computer Science*.

[45] Woo A, Culler D (2003). Evaluation of efficient link reliability estimators for low-power.

[46] Zhou G, He T, Krishnamurthy S, Stankovic JA (2006). Models and solutions for radio irregularity in wireless sensor networks. *ACM Transactions on Sensor Networks*.

[47] Bachir A, Barthel D, Heusse M, Duda A Hidden nodes avoidance in wireless sensor networks.

[48] Schoellhammer T, Greenstein B (2006). Hyper: a routing protocol to support mobile users of sensor networks.

[49] Koksal CE, Balakrishnan H (2006). An abstraction layer for neighborhood discovery and cross-layer metrics. *IEEE Journal On Selected Areas in Communications*.

[50] Zhang H, Arora A, Sinha P (2009). Link estimation and routing in sensor network backbones: beacon-based or data-driven?. *IEEE Transactions on Mobile Computing*.

[51] Zhang H, Sang L, Arora A (2010). Comparison of data-driven link estimation methods in low-power wireless networks. *IEEE Transactions on Mobile Computing*.

[52] Vasudevan S, Adler M, Goeckel D, Towsley D (2013). Efficient algorithms for neighbor discovery in wireless networks. *IEEE/ACM Transactions on Networking*.

[53] Chen BB, Hao S, Zhang M, Chan MC, Ananda AL DEAL: discover and exploit asymmetric links in dense wireless sensor networks.

[54] Heidemann J, Estrin D (2007). Centralized routing for resource-constrained wireless sensor networks.

[55] Al-Bahadili H (2010). Enhancing the performance of adjusted probabilistic broadcasting in MANETs. *The Mediterranean Journal of Computers and Networks*.

